# Drivers of spatio-temporal changes in paddy soil pH in Jiangxi Province, China from 1980 to 2010

**DOI:** 10.1038/s41598-018-20873-5

**Published:** 2018-02-09

**Authors:** Xi Guo, Hongyi Li, Huimin Yu, Weifeng Li, Yingcong Ye, Asim Biswas

**Affiliations:** 1 Key Laboratory of Poyang Lake Watershed Agricultural Resources and Ecology of Jiangxi Province, Jiangxi Agricultural University, Nanchang, 330045 China; 2 Department of Land Resource Management, School of Tourism and Urban Management, Jiangxi University of Finance and Economics, Nanchang, 330013 China; 3 School of Environmental Sciences, University of Guelph, Guelph, ON N1G 2W1 Canada

## Abstract

The spatio-temporal distribution soil pH is critical for understanding the productivity and long-term sustainability of our agri-ecosystem. This study quantified the spatio-temporal distribution of paddy soil pH in Jiangxi province, China, and the potential driver of the change between 1980 and 2010. Data from the Soil Survey Information of Jiangxi province (1980s) and Jiangxi Soil Testing and Fertilizer Recommendation study (2010s) were collected and categorized into six pH ranges from strongly-acidic to strongly-alkaline with unit pH differences. Changes were calculated from the maps developed using the Pedological Knowledge base for 1980s data (without geolocation) and geostatistical methods for the 2010s data (geolocated). An overall 0.6-unit decrease and a major shift of soil pH from weakly-acidic (54% → 18%) to acidic (35% → 74%) was observed over the province in a scattered fashion with concentration in the central part and the Poyang Lake area. About half of the area under paddy cultivation went through acidification by at least one pH unit and 7% by at least 2 pH units, while 40% of the area remained unchanged. Excessive fertilizer application and acid-rain intensity contributed to the acidification. Thus, a more knowledge-based and comprehensive fertilizer management should be adopted to make paddy production sustainable in the province.

## Introduction

Soil, a finite and non-renewable natural resource, plays a key role in the provision of ecosystem services through food, fiber and fuel production, climate regulation, biodiversity conservation, freshwater supply, habitat for living organisms, cultural heritage, construction and infrastructure, and energy sustainability and to face the global challenges^1,2^. Therefore, it is of utmost importance to not only maintain but also to improve our natural resource for a sustainable future. However, the highly dynamic nature of soil conditions make this a substantial challenge^3,4^. Moreover, climate change and the anthropogenic pressures from an ever-increasing global population are resulting in tremendous pressures^5,6^. Changes in soil conditions are connected to soil physical health, soil organic matter levels, soil erosion, compaction, acidification, salinization, desertification, pollution, and soil nutrient imbalances to the services provided (e.g. agricultural production and ecosystem health)^7^.

Soil acidification alters the biogeochemical cycles and adversely affects ecosystem functioning by altering soil conditions^3,4^. Naturally, soils are strongly buffered by the minerals/ions released through weathering; thus, natural soil acidification may occur very slowly over hundreds to millions of years^8^. However, soil acidification became a major problem in intensive agricultural systems and it has been reported worldwide mostly attributed to the use of N-fertilizers^4,7,9–12^. For example, the Chinese agricultural system has been highly intensive since the early 1980’s from the use of large quantities of chemical fertilizers and an abundance of other input resources. China consumed about 30% of the global fertilizer in 2014 (193.29 million tonnes) and applied on average 565.25 kg of fertilizer ha^−1^ of arable land compared to the global average of 138.04 kg ha^−1 ^^13^. In terms of fertilizers, N-fertilizers were consumed in much larger quantities than others (31.08 million tons in China and 108.94 million tonnes in the World in 2014) and the rate of application is extraordinarily high in certain regions^10,12,14–18^ compared to other parts of the world, such as North America and Europe^10,14^. Higher amounts of fertilizer consumption have already degraded soils and affected the environmental quality in various parts of China^10,12,14–20^, including the North China Plain^15^, black soil of North-East China^21^, the red soil area^22^, and the Taihu Lake area^14^. This may also have a direct and an indirect influence on soil acidity^10,16,23^. Additionally, the effect of acid rain in the region also contributed to the acidification^24–28^. However, anthropogenic soil acidification has received less attention while information on the change is crucial for monitoring soil conditions and in assessing its function in a sustainable ecosystem^7^. The changes in soil acidity in Chinese croplands have been assessed at the regional-1^29^ to the national-scale^4^ but without any spatial information which is crucial for developing regionalized management strategies and policies. Spatial variation of soil acidity along with other properties were characterized using a digital soil mapping approach at various scales^20,30–34^. There are studies that quantified the spatial and temporal changes of soil properties, including soil carbon and organic matter^31,32^, soil nutrients^19,35^, and soil moisture^36,37^. Spatio-temporal changes in soil acidity have been quantified using legacy soil test data at the national scale in South Korea^7^ and at regional scales in England and Wales^38^, and in Victoria, Australia^9^. However, the spatio-temporal changes in soil acidity are rarely addressed in China, particularly at the provincial scale^39^.

Jiangxi is a major agricultural province in central China with an arable land area of around 28,000 km^2^, among which paddy (rice) production covers an area of about 22,000 km^2^. Rice planting history in this region goes back 12,000 years and the area remains one of the major national grain growing regions in China to the present day^40^. The Poyang Lake plain (considered to be the origin of rice) and the lower Gan and Xiu valleys are still the major rice growing regions in the province. Jiangxi contributes a major portion (~23 million tons) of China’s total rice production (208.239 million tons compared to 741.477 million tons globally in 2014), which has tripled over last five decades mainly from new high-yielding hybrid varieties, improved crop management practices, and the use of fertilization and irrigation^17^. However, a very slow rise, or almost stagnation, was observed in rice production since the late 2000s^13^ in spite of technological advancements, varietal development, and plant protection success. The quality of the soil as affected by anthropogenic changes was identified as a major contributor along with others^17^. Though rice can grow both under upland and submerged soil conditions, the most widespread practice of rice cultivation in the province of Jiangxi is submerged, which accelerates a range of chemical reactions as affected by soil pH and determines the availability of nutrients and affects rice production^41^. Therefore, pH is a crucial factor in the quality assessment for paddy soils.

The objective of this study was to quantify the temporal change in the spatial variability of soil pH within the province of Jiangxi over the last 30 years and to identify the possible drivers for the change. The spatial and temporal distribution of soil pH at the province-scale will indicate the status, and help identify hotspots for the development of Jiangxi soil acidification prevention and control measures.

## Results

### Spatio-temporal changes of soil pH

#### Exploratory data analysis

The average pH value for the 1980’s (5.8) decreased by 0.6 units during the 2010’s period (5.2) (Table 1). The change was also observed in the distribution of samples representing different pH ranges. For example, almost all the soil samples collected during the 1980’s were between pH 4.5 and 8.5 (pH <4.5 represented 0.6% of the samples) with the majority between pH 4.5 and 6.5 (represented 89% of samples) (Table 1). This means that most of the soil samples collected and measured during the 1980’s were between weakly acidic (54% of samples) to acidic (35% of samples) followed by neutral (5.5% of samples). However, the majority of the soils collected during 2010’s were between pH 4.5 and 5.5 (73.9% of samples) indicating the dominance of acidic soil pH in the province. More than double (35% → 74%) the proportion of soil samples collected during 2010’s within this pH range (4.5 and 5.5) over the 1980’s period contributed to the decrease in the proportion of soils with pH between 5.5 and 8.5. Overall, the proportions of soil samples with pH between 5.5 and 8.5 decreased while the proportion of soil samples with pH <5.5 increased during the 2010’s period over the 1980’s period (Table 1). This provides a clear indication of the general shift of soil pH towards more acidic over the past 30-years in the province^4,21^. A similar decrease in soil pH was also reported from other provinces of China including Guangdong^42^, Guizhou^43^, Henan^44^, Jiangsu^45^, Laioning^46^, and Zhejiang^47,48^. There were no soil samples with pH > 8.5 (weakly alkaline) recorded in the province at any time.

**Table 1:** Number (and percentage of the total) of samples in each grades of soil from the province of Jiangxi, China at two separate times.

Years	Number of samples (Percentage)						Average pH value
	Total	Weakly Alkaline (8.5 > pH > 7.5)	Neutral (7.5 > pH > 6.5)	Weakly acidic (6.5 > pH > 5.5)	Acidic (5.5 > pH > 4.5)	Strongly acidic (4.5 > pH)	
1980s	1,154	17 (1.5%)	110 (5.5%)	623 (54.0%)	404 (35.0%)	5 (0.4%)	5.8
2010s	10,155	69 (0.7%)	283 (2.8%)	1,795 (17.7%)	7,508 (73.9%)	500 (4.9%)	5.2

#### Spatial distribution of soil pH at two separate times

**Figure 1:**
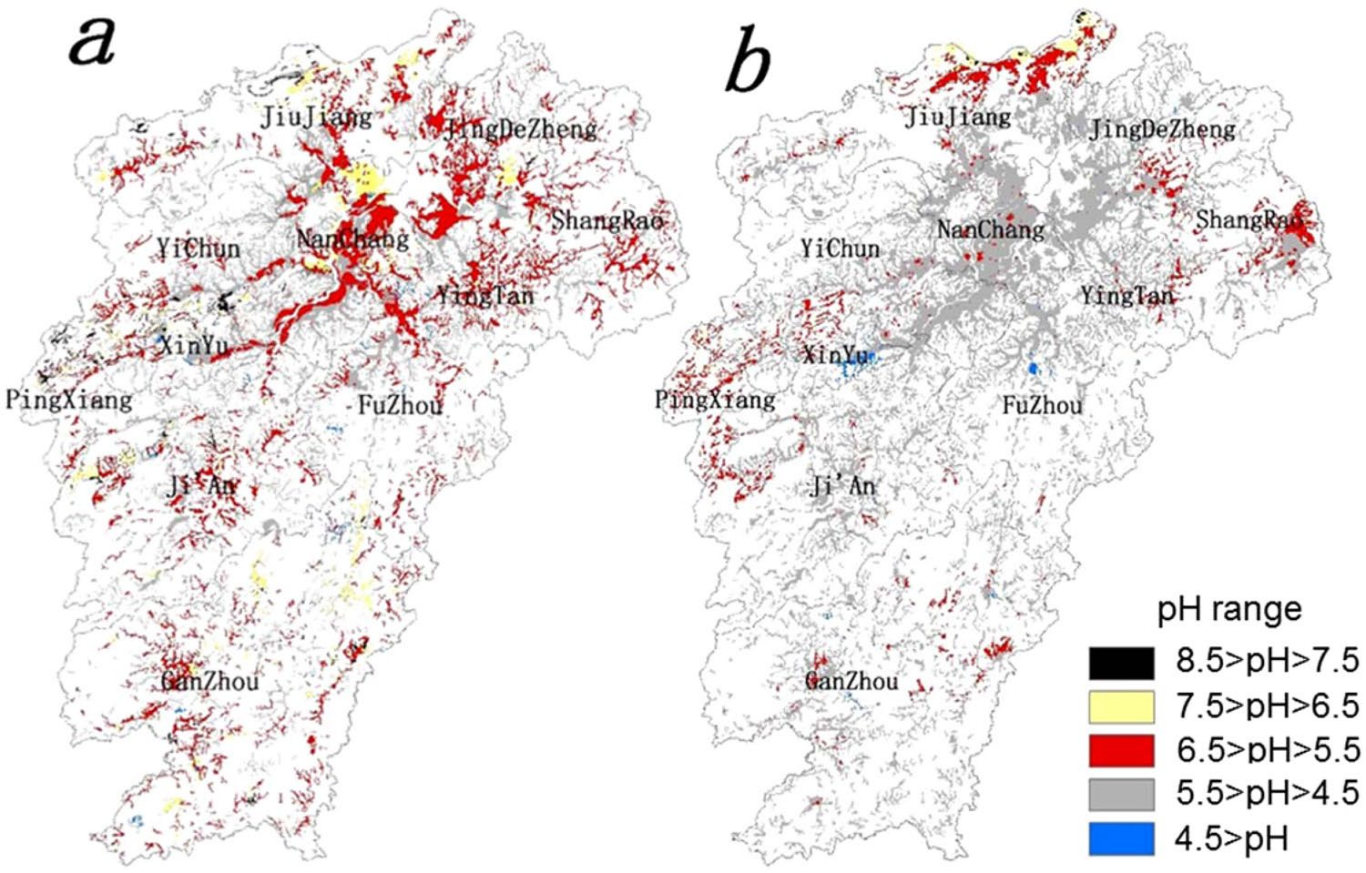
Spatial distribution of paddy soil pH ranges within the province of Jiangxi during the (**a**) 1980’s and (**b**) 2010’s period. Internal subdivisions indicate divisional boundaries with appropriate names. White area indicates land not under paddy cultivation. The figure was prepared in ArcGIS version 10.4 (ESRI Inc., Redlands, CA, USA, http://www.esri.com/products).

**Figure 2:**
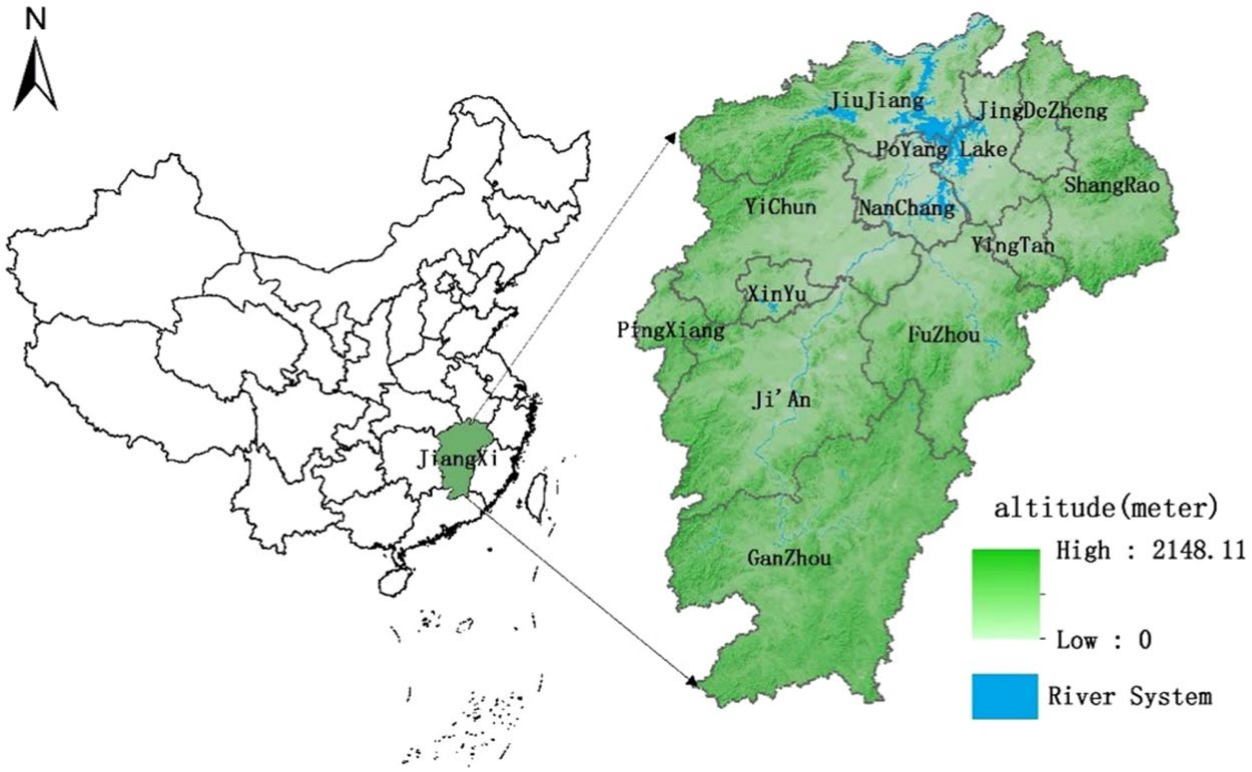
Geographic location with relative elevation map of Jiangxi Province. The administrative division names are indicated on the map. The figure was prepared in ArcGIS version 10.4 (ESRI Inc., Redlands, CA, USA, http://www.esri.com/products).

The spatial distribution of soil pH in the 1980’s clearly showed a scattered distribution of weakly acidic soils (6.5 > pH > 5.5) throughout the province with a dominance around the the center mainly surrounding the Poyang Lake area (Fig. 1a). South of the lake and along the Ganjiang and Fuhe rivers (Fig. 2) were mainly dominated by weakly acidic soils (6.5 > pH > 5.5). The second dominant acidic soils (5.5 > pH > 4.5) were also present all over the province with some concentration in the southern and western parts of the province. Some neutral soils (7.5 > pH > 6.5) were found around the west side of the Poyang Lake and rarely in the southern part of the province while a small area with weakly alkaline soil (8.5 > pH > 7.5) was found in the northwestern part of the province (Fig. 1a). In contrast, the province was mainly dominated by acidic soils (5.5 > pH > 4.5) during the 2010’s period with some scattered areas of weakly acidic (6.5 > pH > 5.5) soils concentrated in the northern, western and eastern boundary of the province (Fig. 1b). Some areas with neutral soils (7.5 > pH > 6.5) were also concentrated mainly in the northern boundary of the province. A few areas with strongly acidic soils (4.5 > pH) were found upstream in the Ganjiang and Fuhe rivers. Visual comparison of the maps of the 1980’s period (Fig. 1a) and the 2010’s period (Fig. 1b) showed a clear change in the distribution pattern of soils with various levels of acidity. The change from weakly acidic (35% of samples) to acidic (73.9% of samples) soils (Table 1) were very clear with dominating acidic soils in the center part of the province (Fig. 1). A similar change in spatial distribution of soil pH in general as well as in paddy soil also observed in other parts of China including the province of Guangdong^42^, Guizhou^43^, Henan^44^, Jiangsu^45^, Liaoning^46^, and Zhejiang^48^.

#### Temporal change of soil pH

**Figure 3:**
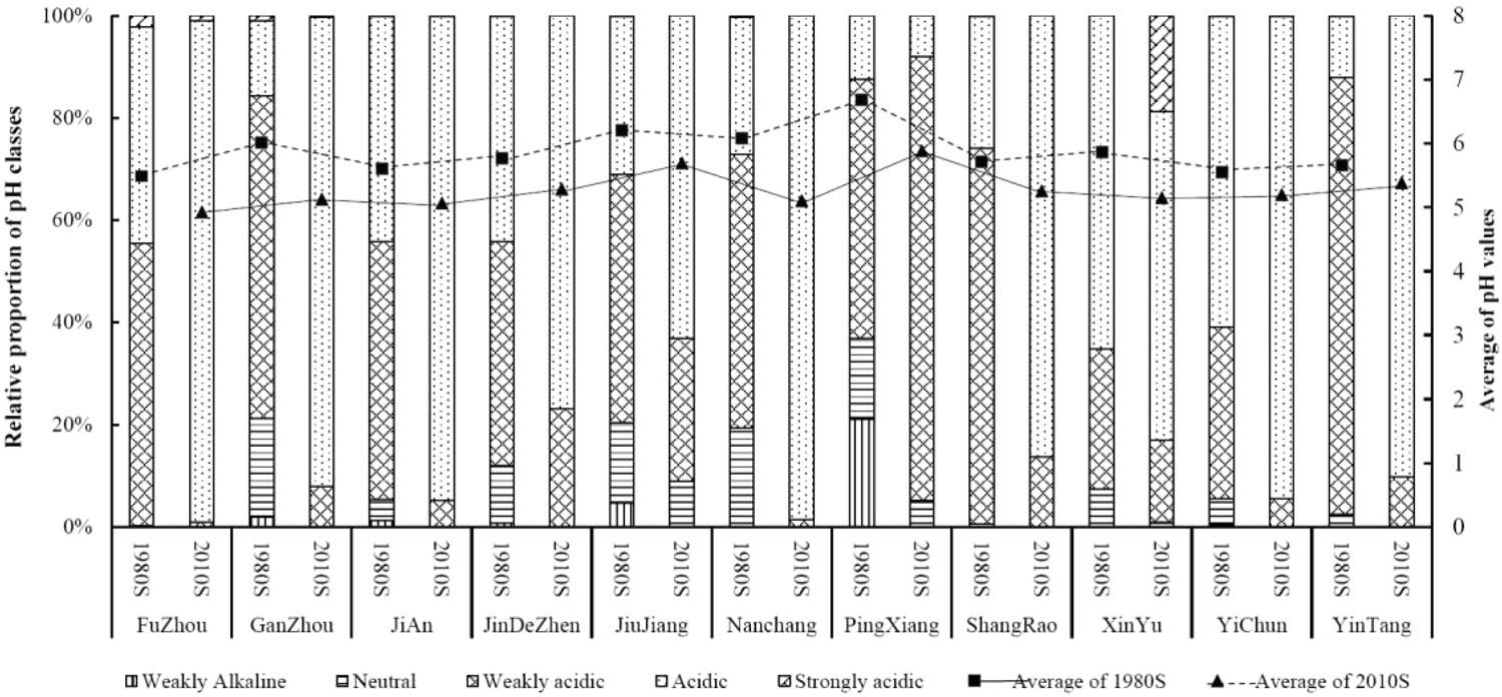
Change in proportions of soil pH grades at 11 divisions within Jiangxi Province, China from 1980s to 2010s. The Figure was prepared in Microsoft Excel.

An increasing trend of the proportion of the acidic soils (5.5 > pH > 4.5) while a decreasing trend of the proportion of weakly acidic soils (6.5 > pH > 5.5) were observed for 10 out of 11 divisions (except PingXiang) over the past 30-year period (Fig. 3). The proportional increase of the soil samples with pH between 4.5 and 5.5 was a result of the proportional decrease of soils with pH ranges between 5.5 and 8.5. This indicated a shift in the areas with more acidic soils (decrease in soil pH or increase of soil acidity) at the divisional levels over the last 30 years. On the contrary, PingXiang showed an opposite trend where the proportions of the weakly acidic (6.5 > pH > 5.5) soils increased. A decrease in the proportion of soil samples with pH ranged between 6.5–8.5 and 4.5–5.5 were also observed for this division. Only one division (XinYu) showed an increase in the proportions of the strongly acidic (4.5 > pH) soils (Fig. 3). However, an overall decreasing trend in the average pH of all divisions was observed from 1980’s to 2010’s (Fig. 3 and Table 2). Similar trend was observed in other provinces including Guangdong^42^, Guizhou^43^, Henan^44^, Jiangsu^45^, Liaoning^46^, and Zhejiang^48^. The average pH of the soils of the province decreased to 5.23 during the 2010’s from 5.84 in the 1980’s with an overall decrease of 0.61 units of pH. The largest decrease (0.98 unit of pH) was observed for the division of NanChang, while the smallest decrease was observed for YiChun (0.36 unit of pH) (Table 2). The decrease in the soil pH indicated the increase in the soil acidity at divisional levels. A small decrease in the overall area under paddy cultivation was observed for almost all the divisions during the last 30 years (Table 2).

**Table 2:** Average and the change of soil pH and the area of paddy cultivation at divisional level at two separate times in Jiangxi Province.

Cities	1980s		2010s		Change in Average pH
	Area (km^2^)	Average pH value	Area (km^2^)	Average pH value	
GanZhou	3195.220	6.02	3173.355	5.13	0.89
NanChang	2428.733	6.09	2417.559	5.11	0.98
YinTan	622.317	5.86	618.076	5.39	0.47
ShangRao	3493.828	5.72	3466.032	5.26	0.46
Ji’An	3061.132	5.60	3040.272	5.07	0.53
FuZhou	2105.192	5.49	2090.567	4.93	0.56
PingXiang	380.946	6.69	377.322	5.89	0.8
JiuJiang	2935.445	6.21	2912.289	5.70	0.51
JingDeZheng	879.244	5.78	873.268	5.29	0.49
XinYu	552.550	5.87	548.785	5.15	0.72
YiChun	3207.063	5.56	3185.165	5.20	0.36
Total	22861.670	5.84	22702.690	5.23	0.61

**Figure 4:**
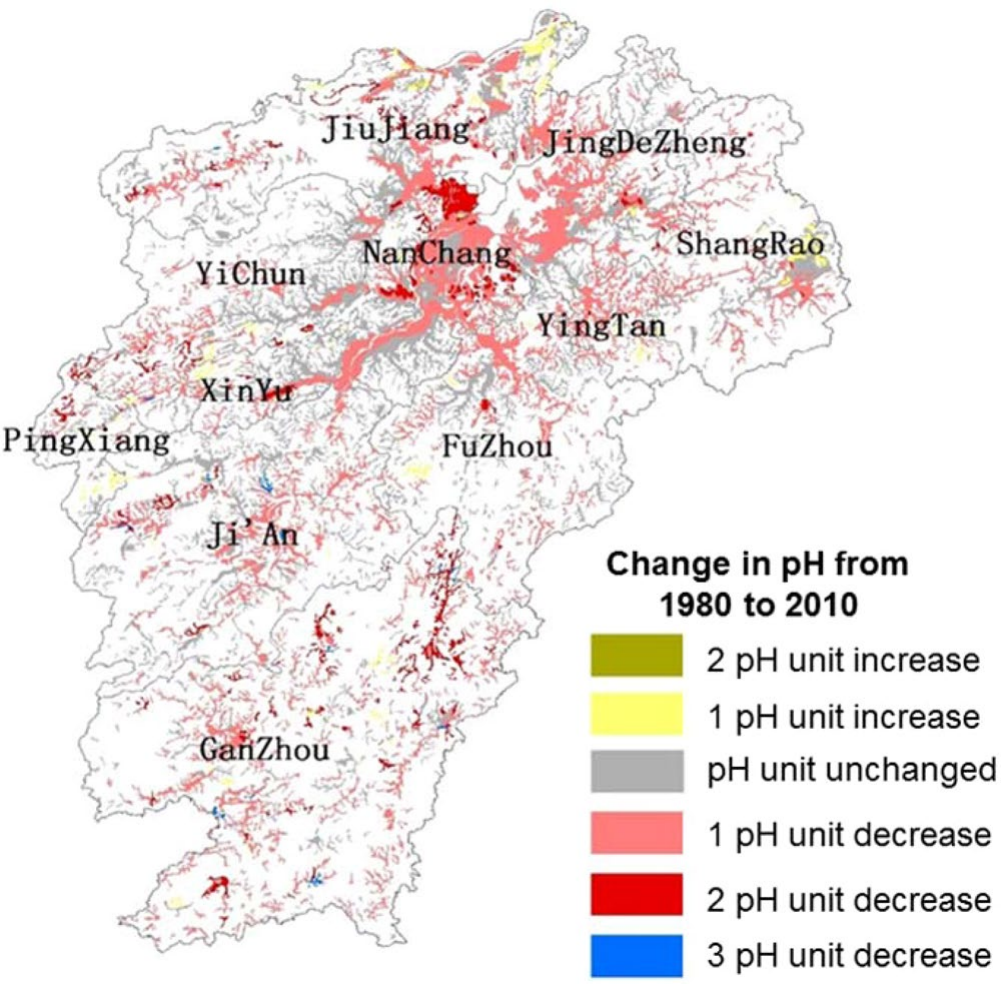
Spatial distribution of the change of pH grades of paddy soils in Jiangxi Province, China. Internal subdivisions indicate divisional boundaries with appropriate names. The figure was prepared in ArcGIS version 10.4 (ESRI Inc., Redlands, CA, USA, http://www.esri.com/products).

The pH of about 40% of the paddy soil area within the province did not show any change in pH range over the last 30 years (Table 3). However, more than half of the area (50.3%) under paddy cultivation showed at least one-unit decrease in pH range. The change in one-unit pH was scattered all over the province (Fig. 4) with major concentrations around Poyang Lake and along the Ganjiang and Fuhe rivers (Fig. 2). A scattered distribution near cities also showed a unit pH change. There were about 7% of the paddy soil area that showed at least two-unit pH decrease (Table 3). These locations were concentrated mainly along the southern side of the Poyang Lake and Nanchang city with some scattered locations in the southern part of the province (Fig. 4). Dropping two units of soil pH within 30 years could be a major issue in terms of soil functioning, yet a considerable 7% (Table 3) of the paddy soil area exhibited the change. Extreme drops of 3-unit pH was also observed but in small areas (0.4% of the total area) (Table 3 and Fig. 4). A minimal (2.8%) proportion of the total area showed an increase in soil pH values by at least one unit (Table 3). These areas were concentrated along the northern and eastern borders of the province with some scattered locations in the south to south central areas and the western borders (Fig. 4).

**Table 3:** Change in pH ranges and the area of change of paddy soil pH within Jiangxi Province, China.

pH Change from 1980 to 2010	Area (km^2^)	Percentage (%)
2 pH unit increase	1.133	0.00
1 pH unit increase	638.258	2.81
pH unit unchanged	8,954.199	39.44
1 pH unit decrease	11,412.912	50.27
2 pH unit decrease	1,604.729	7.07
3 pH unit decrease	91.459	0.40
Total	22,702.690	100

#### Center of gravity of the pH changes

**Figure 5:**
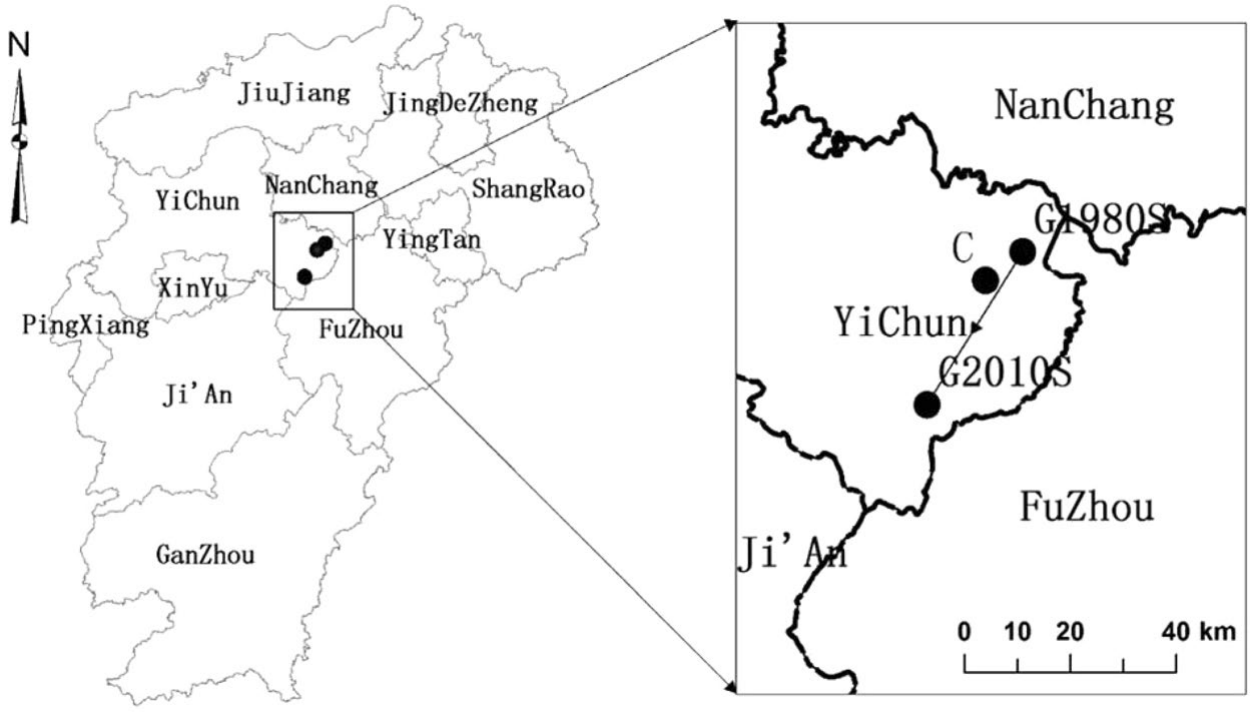
Change in center of gravity for the soil pH from 1980s to 2010s along with polygon center of gravity in the Jiangxi Province, China. Internal subdivisions indicate divisional boundaries with appropriate names. The figure was prepared in ArcGIS version 10.4 (ESRI Inc., Redlands, CA, USA, http://www.esri.com/products).

The position of the gravity center was calculated based on the distribution of the pH values in the 1980s and 2010s. The geolocation for the center during the 1980s period was G1980S (115.858863E, 27.981985N) and during the 2010s was G2010S (115.84682E, 27.91672N) (Fig. 5). Both centers were in the YiChun division. A clear shift of the center of about 40 km in the southwest direction was observed over the last 30-year period. This indicated that the overall trend in acidification was towards the southwestern direction and the speed of change was about 1.33 km per year. However, these gravity centers were different from the polygon center of gravity (C, Fig. 5) and indicated an uneven distribution of the paddy soil pH in the province.

### Drivers of soil acidification

**Figure 6:**
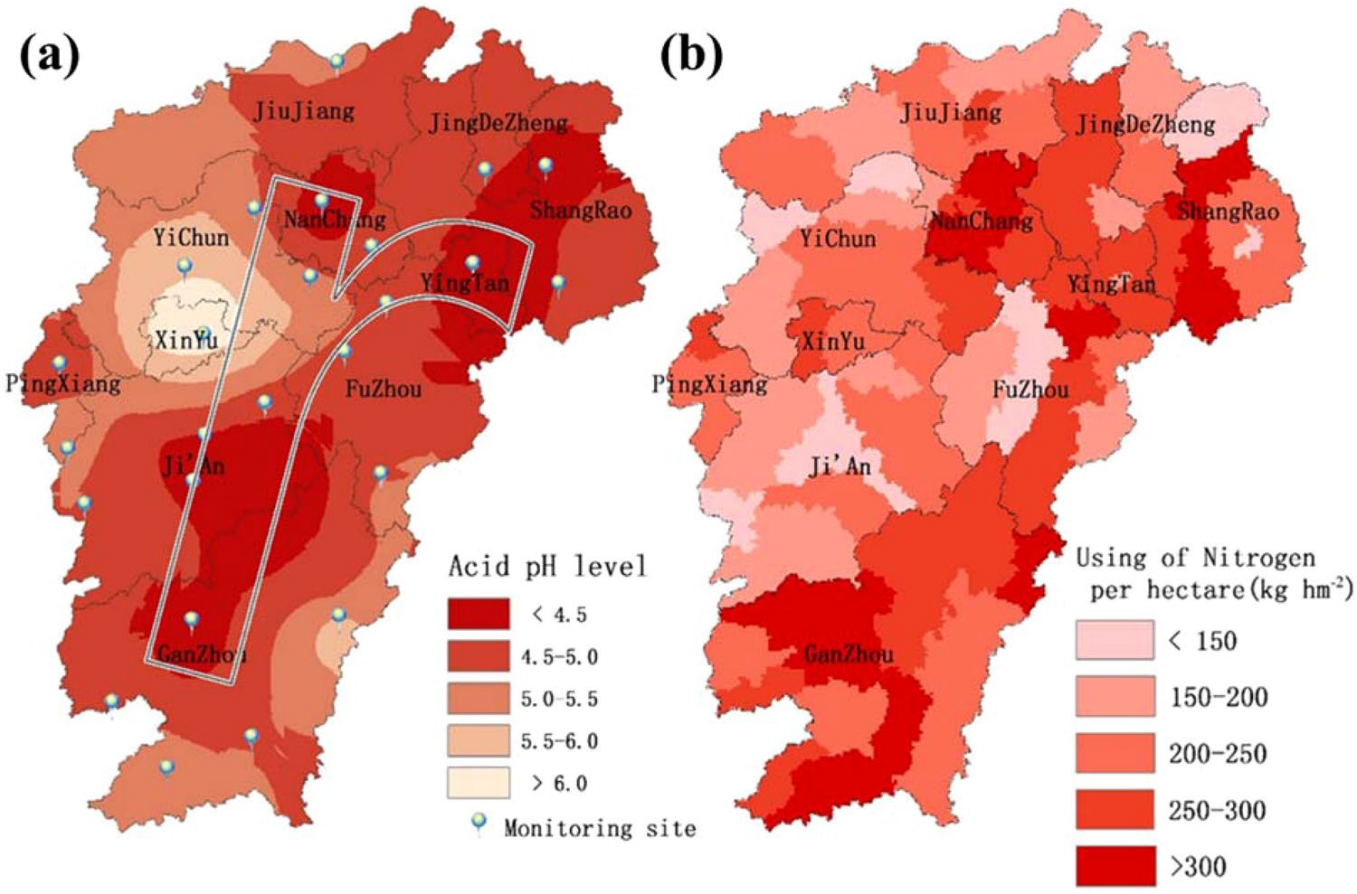
Spatial distribution of (**a**) acid rain levels and (**b**) nitrogen application rate per hectare within the province of Jiangxi, China. Internal subdivisions indicate divisional boundaries with appropriate names. The figure was prepared in ArcGIS version 10.4 (ESRI Inc., Redlands, CA, USA, http://www.esri.com/products).

The spatial distribution of average acid rain data over 1985 to 1990 showed a pattern; a decrease in acidity was observed form the east to west in the upper half of the province while an increase was observed from the north to the southern border (Fig. 6a). Please note that the acid rain data used in this study were only between 1985 and 1990 and not the whole study period. The acidity value gradually increased from the center to the eastern and western borders in the southern half of the province. Average pH of acid rain as low as 4.17 was observed in the YingTan division followed by NanChang. The highest average pH of acid rain was observed in the XinYu division. The acid rain drop frequency (occurrences of rain water with acidic pH) was the highest in the GanZhou division reaching to 98.9% followed by YingTan and Ji’An at 97.0% and 96.7%, respectively (data not shown). These two values (acid rain pH and acid rain drop frequency) were then combined to delineate acid rain intensity and the severe acid rain region, which looked like small English letter ‘r’ (Fig. 6a). The areas along a straight line from NanChang to GanZhou through Ji’An and the slash over Ji’An, FuZhou, YingTan and ShangRao divisions were identified as the severe acid rain region. A similar trend in acid rain intensity in the province was reported previously^24–27^.

The spatial distribution of the average Nitrogen fertilizer application rate based on 30 years of data at the county and district levels indicated highly variable application rates between 70 kg ha^−1^ to over 400 kg ha^−1^ (Fig. 6b). Very high application rates were observed in areas within the divisions of NanChang, ShangRao, GanZhou and FuZhou, while the lowest rate was observed in areas within the divisions of Ji’An, FuZhou, YiChun and ShengRao (Fig. 6b). However, studies identified a required rate of Nitrogen fertilizer for high yielding paddy varieties in Southern China to be between 126 and 236 kg ha^−1^ with an optimum rate between 104 and 192 kg ha^−1^ while the ecological rate was identified between 69 and 115 kg ha^−1 ^^49^.

The linear relationship showed a reasonable agreement (R^2^ = 0.708 with p value < 0.01) between the change in soil acidification and the acid rain intensity and the Nitrogen fertilizer application rate. The change in soil pH = 0.585× average N application rate ha^−1^ +0.553× acid rain intensity −0.12. This indicated the possible influence of acid rain intensity and Nitrogen fertilizer application rates on soil acidification in the province of Jiangxi. A low Variance Inflation Factor (VIF) value (1.769) indicated the absence of collinearity between acid rain intensity and fertilization rates and random values of residuals indicated the absence of any trends in the data.

## Discussion

Soil acidification as measured by the change in level of soil pH or the logarithm of hydrogen ion activity in soil, is an important soil health indicator and determines the soil’s productivity and other ecosystem services^50,51^. It affects the soil’s physical, chemical and biological processes as well as plant growth and production and can have a direct and indirect influence on soil functioning. Among many others, scorching effect, increasing solubility and toxicity of various metal ions (e.g. Al, Mn), unavailability through fixation (e.g. P, Fe, Mo, Cu, Zn, B) or deficiency through changing ratios (e.g. Ca, Mg), deteriorating environmental conditions, decreasing microbial activity and crop productivity are few examples of its effect^8,51,52^. Soil pH may change naturally but at a very slow pace and may not have a strong influence due to the adaptation of the ecosystem^53^. However, the accelerated change of soil pH may result from massive anthropogenic influences on the ecosystem. Intensive agricultural practices had the strongest influence on accelerated soil acidification^3,4,12,14,18,29,39,53^.

Since the 1980’s, the Chinese agriculture has been intensified greatly through modern practices, input of large amounts of chemical fertilizers, irrigation and other resources^17,49^. Adaptation of inappropriate cropping systems and the application of large quantities of Nitrogen fertilizer lead to low fertilizer use efficiency and contributed substantially to soil acidification in China^4,10,12,14–19,23,35,49,54–57^. Jiangxi province is no different^40,54,58^. An overall decrease of 0.6 units of paddy soil pH over the last 30-year period (Table 1) in the Province of Jiangxi might be the result of the intensification of agriculture from increased population pressure^40,54^. Similar situation was reported for other provinces in China including Guangdong^42^, Guizhou^43^, Henan^44^, Jiangsu^45^, Liaoning^46^, and Zhejiang^48^. Rice is the staple food of the province of Jiangxi and covers the majority of the cultivated areas^40^. Due to the logarithmic nature of pH measurements and expression, a decrease of about 0.3 units of pH doubles the hydrogen ion activity in soil^51,52^. A total of 0.6 units over 30 years or about 0.2 unit per 10 years indicated a significant increase in hydrogen ion activity per decade in the paddy soils of Jiangxi, which will have strong influence on soil functioning. A decrease of 0.3 to 0.8 units of pH over about 20 years under cash crop systems was also reported by Guo, *et al*.^4^ at the national scale. A similar decrease over 2 to 5 decades was observed in other provinces including a decrease of about 0.5 unit from the semi-arid Tibetan Plateau^18^, 0.49 unit from Liaoning province^46^, 0.4 unit from Guangdong province^42^, and 0.25 unit from Zhejiang province^48^.

Over the last 30 years, the area with acidic soils increased from about one-third (35%) to three-fourths (73.9%) of the total area under paddy cultivation. However, a large decrease in the area under weakly acidic soil (from 54% to 17.7%) was observed over the province in this period. The decrease in area under weakly acidic to an increase in the area under acidic soil clearly indicated a shift in the soil acidity and its functioning^4^. At least a unit pH change was observed for more than half of the area under paddy cultivation. The changes were observed all over the province with some concentration around the central part (Fig. 4) having less topographical variations (Fig. 2). This may be from the abundance of freshwater from the Poyang Lake and suitability of paddy cultivation in the flatter central part of the province that might have intensified the agricultural practices leading to greater changes in soil acidification^40,54,58^. A similar trend was observed in Jiangsu^45^ and Zhejiang^48^ province, where the largest change was observed in the lower topographic regions including plateau and surrounding lake area (e.g. Lixiahe plain, Taihu lake). A drop of two-unit pH was also observed around the lake (Fig. 4) indicating about 100 times more activity of hydrogen ions in soil^51,52^. However, a more detailed study covering various factors of paddy production can only quantify the effect comprehensively.

Paddy soil acidification is a complex process and can be influenced by natural, artificial or anthropogenic factors^4,12,28,42–48,55–57,59–62^. A good agreement was observed between the change in soil acidification with Nitrogen fertilizer application and acid rain intensity in all regions of the province. In general, the influence of acid rain was stronger in the southern part of China with higher rainfall and the quality of air (affected by industry) leading to acid-rain mainly from carbon dioxide^18,24–28,54,59,61,62^. Our result was in line with the general trend of the effect of acid rain intensity. The Nitrogen fertilizer application rate also showed a very similar effect on the soil acidification in the study area^4,10,12,15–19,23,35,49,54,57^. From a long-term fertilization experiment, Zhang^29^ reported that unreasonable agricultural practices such as Nitrogen fertilizer application intensified the soil acidification process over other activities, such as burning of fossil fuels contributing acid-deposition. Meng, *et al*.^63^ reported the greatest effects of Nitrogen fertilizer on farmland soil acidification from long-term monitoring. In the current study, Nitrogen fertilizer application rates in Jiangxi province had a slight stronger influence on soil acidification over acid rain (determined from regression coefficients) and was consistent with other studies^4,23,29^. However, true effect of acid rain intensity can only be examined by taking the integrated data over the whole study period and was a limitation for this study. Zhou *et al*.^64^ reported that in addition to excessive chemical Nitrogen fertilizer application leading to soil acidification, the decline in the use of organic fertilizer also caused soil acidification. In addition, the soil acidification may also have been affected by different soil types with variable buffering capacity and responses to such external factors as climate, topography or cropping system and management practices leading to the spatial difference of soil acidification^18,22,53,59^. The effect of carbon and nitrogen cycles in different plant systems, soil respiration leading to carbonate leaching and unreasonable administration of straw, manure, etc. may also have a strong effect on soil acidification^59,64^. While this study quantified the spatio-temporal variability of paddy soil pH and its relationship with acid-rain intensity and Nitrogen fertilization, future studies should focus on quantifying the spatio-temporal effects of other factors at different scales (city, country, province) using the county level land fertility evaluation results combining with local environmental monitoring data, planting characteristics and topography.

The changes in soil acidification as affected by anthropogenic factors may appears mainly from the application of Nitrogen fertilizers and acid rain intensity affecting the environmental quality in the study area^11^. Due to low use efficiency, the majority of the fertilizer nutrients are released into the environment. An optimum Nitrogen fertilizer application rate may maintain, or even improve, production while significantly reducing the environmental impact, including soil acidification^4^. This study clearly showed the areas of change that are critical and require attention. Based on the status and the trend in acidification, a more knowledge-based and comprehensive agricultural management practices should be adopted to make rice production sustainable throughout the province as well as maintaining the provincial contribution to China’s rice production.

## Materials and Methods

### Study Area

Jiangxi province is located in the central region of China (latitude 24°29′–30°40′ longitude 113°34′–118°28′) and at the south coast of the Yangtze River (Fig. 2). The province covers an area of about 166,900 km^2^ spanning about 620 km in the north-south and about 490 km in the east-west direction. Poyang Lake, the largest freshwater lake in China, is located at the northern part of the province surrounded by mountains on three sides. Generally, the center of the province is situated at low altitudes and surrounded by high altitudes around the perimeter with a basin like opening in the south (Fig. 2). The interior area of the province has a warmer climate with an annual average temperature of 16.4~19.4 °C (temperature difference from north to south is about 3 °C). Outer reaches of the province have a mild and humid subtropical climate with abundant rainfall and a long non-frost period. The average annual rainfall is ~1,300–2,000 mm with uneven distribution between seasons and regions. The northeastern part of the province receives the highest amount of rain and north of Jiujiang (located in the north-west of the province) receives the lowest amount of rain. The main food crop is rice followed by sweet potato, wheat, rapeseed, and tea. The major soil types include red soil, paddy soil, yellow soil and mountain yellow brown soil (Following Chinese System of Soil Classification). Paddy soils mainly include lake and river sediments, quaternary red clay and acidic crystalline weather class (in hilly areas) and is widely distributed throughout the province with three major subclasses; gleied (most common), waterlogged and flooded^65^.

### Data Sources

Soil pH data from two studies were collected from two separate periods; a) Soil Survey Information of Jiangxi Province carried out during the 1980s (identified as the 1980s period) and b) Jiangxi Province Soil Testing and Fertilizer Recommendation study carried out during the 2010s (identified as the 2010s period). The soil pH data of the 1980s period were collected from “Chinese Soil Species Records”^58^ and “Jiangxi Soil”^65^. There was a total of 172 typical cross-sectional surface pH values and 982 agricultural soil samples pH values from 99 counties throughout the province. Some of these samples were represented as between a range of soil pH and not by absolute values. Therefore, we have used the mid-point of the range of pH values to represent the average pH value. Soil samples from the 1980s period were not georeferenced and the vector files showing the paddy map patches were used to develop a spatially distributed map. Due to some missing vector files showing the paddy map patches during the 1980s, we used vector files from the second land resources survey as the base map. There were a total of 10,155 georeferenced surface (0–20 cm) soil pH values for the 2010s period. A total of 4 to 5 surface (mainly 0–20 cm) soil samples were collected from the selected sites within a 5-m radius area and a composite sample was prepared and the volume was reduced following the National Soil Survey Office Regulation^65,66^. The soil sampling was carried out by the government agency representatives simultaneously from various parts of the province mainly after the harvest of paddy during July-August. Soil pH was measured in the government laboratories on the air dried, ground and sieved (2 mm) soil samples using potentiometric method in 1:2.5 soil:water solution. Details of the pH meters are not available in the report. The pH values from the two periods were used to develop maps (explained later) and to compare the spatial and temporal changes. The fertilizer application data for various regions of the province were collected from the Statistical Yearbook of various counties and districts. The acid-rain data from 30 counties and districts were collected from the “Jiangxi Province Spatial and Temporal Distribution of Acid Rain Research” study performed in the 1990s^24,25^.

### Data Analysis

The soil pH data from two periods were classified following the pH ranges of the second soil survey^58^. The six soil pH ranges were; (1) >8.5- alkaline, (2) 8.5 to 7.5- weakly alkaline, (3) 7.5 to 6.5-neutral, (4) 6.5 to 5.5-weakly acidic, (5) 5.5 to 4.5 -acidic, and (6) <4.5-strongly acidic^66^. Due to a lack of geographic locations for the soil samples collected during 1980s period, we adopted the relatively accurate Pedological Knowledge Based method (PKB)^67^ to characterize the spatial distribution. The PKB principle considered soil data from the same soil type and spatial location of the sampled profiles. Based on the similarity on the parent materials and recorded distribution area, the soil properties of each profile were then connected to the corresponding delineations in the soil maps. In this study, we assigned the sampling points to a Jiangxi 1:250,000 digital soil map and developed a spatially distributed soil pH map of the 1980s period. We used geostatistical analysis based inverse distance weighing (IDW) to interpolate the pH sampling points from the 2010s period. We calculated the temporal change of soil pH from the difference between the soil pH spatial distribution maps from two periods at two spatial scales, at the administrative divisional scales and at the provincial scales by aggregating the data located within the respective boundaries. The divisional level change was expressed as the change in the average pH ranges within each division. As it is not possible to calculate the average of the ranges of soil pH, we have taken the midpoint to represent the range of soil pH and calculated change within a division. For example, we have considered the pH value of 5.0 for the range representing acidic soils with 5.5 > pH > 4.5. The provincial level change was calculated from the total area and the average pH within each division and aggregating them. The areal change of unit pH range was then grouped to express the decrease or increase in soil acidity.

We also calculated the change of the direction of gravity and the area of the soil pH ranges to simulate the trend and direction of soil acidification. The center of gravity was calculated following:\

1 $${\bar{X}}_{t}={\sum }_{i=1}^{n}{S}_{i}{K}_{i}{x}_{i}/{\sum }_{i=1}^{n}{S}_{i}{K}_{i}\,{\rm{and}}\,{\bar{Y}}_{t}={\sum }_{i=1}^{n}{S}_{i}{K}_{i}{y}_{i}/{\sum }_{i=1}^{n}{S}_{i}{K}_{i}$$

where, ${\bar{X}}_{t}\,$and ${\bar{Y}}_{t}$ represented *t* years of soil pH value of the center of gravity coord_*i*_nates *x*_*i*_ and *y*_*i*_, *S*_*i*_ was the corresponding map area and *K*_*i*_ was the corresponding map spot pH range.

To identify the drivers of the pH change, multivariate linear regression was performed between the soil pH values of two periods and the fertilization and acid-rain intensity data for each region. Acid rain intensity was the average of the normalized value pH of the rain and the normalized value of the acid rain drop frequency. Spatial data analysis was performed in ArcGIS (ESRI Inc., Redlands, CA, USA) and statistical analysis was performed in SPSS software (IBM Corp., Armonk, NY, USA).
